# Precocious leaf senescence by functional loss of *PROTEIN S-ACYL TRANSFERASE14* involves the NPR1-dependent salicylic acid signaling

**DOI:** 10.1038/srep20309

**Published:** 2016-02-04

**Authors:** Xin-Ying Zhao, Jia-Gang Wang, Shi-Jian Song, Qun Wang, Hui Kang, Yan Zhang, Sha Li

**Affiliations:** 1 State Key Laboratory of Crop Biology, College of Life Sciences, Shandong Agricultural University, Tai’an, 271018, China

## Abstract

We report here that Arabidopsis PROTEIN S-ACYL TRANSFERASE14 (PAT14), through its palmitate transferase activity, acts at the vacuolar trafficking route to repress salicylic acid (SA) signaling, thus mediating age-dependent but not carbon starvation-induced leaf senescence. Functional loss of *PAT14* resulted in precocious leaf senescence and its transcriptomic analysis revealed that senescence was dependent on salicylic acid. Overexpressing *PAT14* suppressed the expression of SA responsive genes. Introducing the SA deficient mutants, *npr1-5* and *NahG*, but not other hormonal mutants, completely suppressed the precocious leaf senescence of *PAT14* loss-of-function, further supporting the epistatic relation between *PAT14* and the SA pathway. By confocal fluorescence microscopy, we showed that PAT14 is localized at the Golgi, the *trans*-Golg network/early endosome, and prevacuolar compartments, indicating its roles through vacuolar trafficking. By reporter analysis and real time PCRs, we showed that the expression *PAT14*, unlike most of the senescence associated genes, is not developmentally regulated, suggesting post-transcriptional regulatory mechanisms on its functionality. We further showed that the maize and wheat homologs of *PAT14* fully rescued the precocious leaf senescence of *pat14-2*, demonstrating that the role of *PAT14* in suppressing SA signaling during age-dependent leaf senescence is evolutionarily conserved between dicots and monocots.

Leaf senescence is a developmentally programmed dismantling process by which leaf longevity is limited while nutrients are remobilized into seeds as in annual plants or other storage tissues as in perennial plants^1^,^2^,^3^. Leaf senescence starts with an initiation stage during which photosynthetic activities gradually decrease, followed by a degenerative phase when cellular components disassemble while macromolecules degrade, and culminated with a terminal phase when cells die^1^,^2^,^3^,^4^. The onset and progression of senescence affects fitness, yield, and grain quality^4^, thus is under complex control.

Senescence integrates developmental and environmental cues into age-dependent changes in which phytohormones play key roles^1^,^2^,^5^. Salicylic acid (SA), jasmonates (JA), and ethylene promote leaf senescence^6^,^7^,^8^,^9^,^10^,^11^,^12^ while auxin and cytokinins (CK) delay leaf senescence^13^,^14^,^15^. Common execution events downstream of hormone-controlled leaf senescence were postulated^5^ whereas different hormones may influence leaf senescence by changing the age status of plants, by transcriptional changes, or by integrating environmental responses^1^,^2^.

Senescence associates with tremendous transcriptional changes^5^,^16^,^17^,^18^,^19^. Therefore, transcription factors, such as NAC, MYB, bZIP, and WRKY, were most noted for their roles in senescence, whose activities resulted in transcriptional changes of senescence associated genes (*SAG*s)^16^,^17^,^19^,^20^. Despite the extensive studies on transcriptional controls of senescence, important knowledge gaps remain. Senescence by itself is a process of nutrient remobilization from senescing organs to newly initiated leaves or developing grains^4^. Components involved in the dismantling of source tissues and nutrient remobilization to sink tissues are yet to be identified.

We report here the identification of a novel component positively regulating leaf longevity by suppressing SA biosynthesis and signaling. PROTEIN S-ACYL TRANSFERASE14 (PAT14) is one of the 24 Arabidopsis PATs catalyzing the addition of a long chain fatty acid, usually C16 palmitate, to Cys residues thus changing the subcellular localization, activity, or turnover of substrate proteins^21^,^22^,^23^. Functional loss of *PAT14* resulted in precocious leaf senescence that was suppressed by the expression of the wild-type PAT14 but not by its catalytically inactive mutant, suggesting that PAT14 functions through substrate palmitoylation. Transcriptomic studies revealed that *PAT14* loss-of-function accelerated natural senescence without affecting developmental program. In addition, genes involved in SA biosynthesis or signaling were significantly over-represented in the group of upregulated transcripts comparing *pat14* to wild type. Indeed, reducing SA levels or disrupting SA signaling suppressed precocious leaf senescence of *pat14* during development, suggesting that *PAT14* negatively regulates SA biosynthesis or signaling. We further showed that the positive role of PAT14 in leaf longevity is evolutionarily conserved because its maize and wheat homologs fully rescued the mutant phenotype. By using fluorescence probes and pharmacological treatments, we showed that PAT14s are localized at endomembrane compartments along vacuolar trafficking route, namely, the Golgi, the *trans*-Golgi network/early endosome (TGN/EE), and prevacuolar compartments/multivesicular bodies (PVC/MVB), suggesting a key role of vacuole-mediated processes in leaf senescence.

## Results

### Functional loss of *PAT14* results in precocious leaf senescence

**Figure 1:**
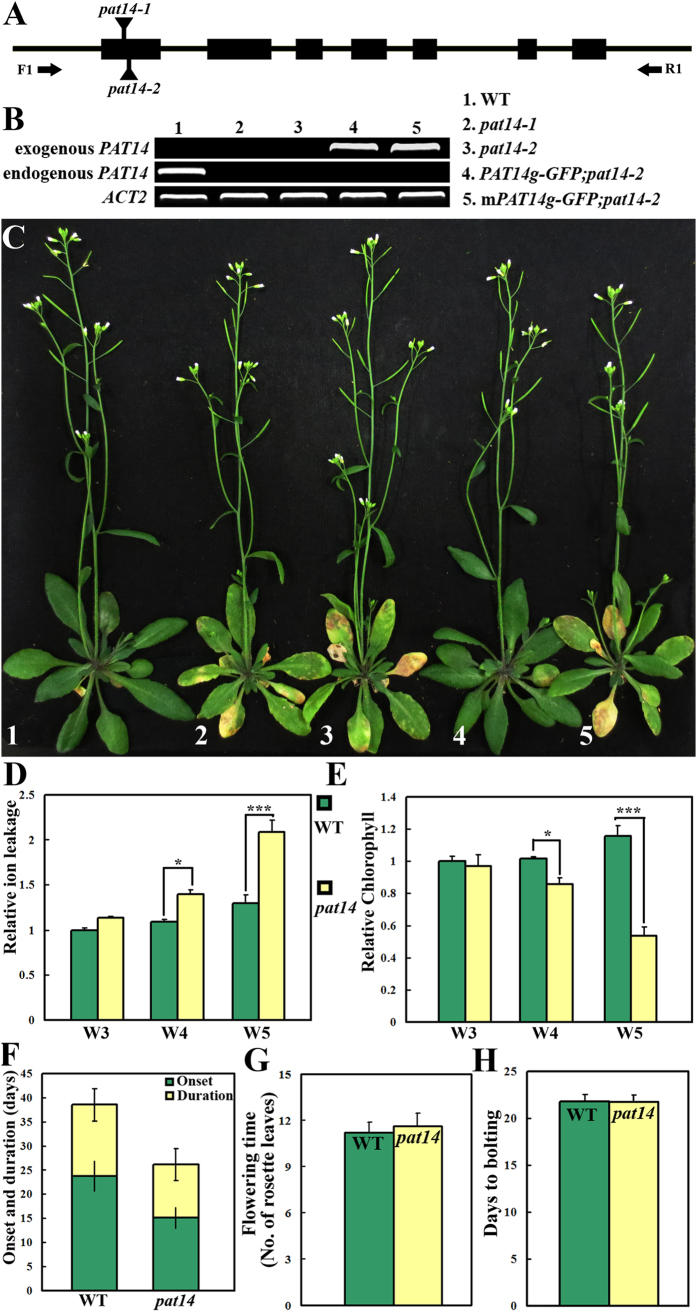
Functional loss of Arabidopsis *PAT14* resulted in early senescence. (**A**) Schematic illustration of the *PAT14* genomic locus and the two T-DNA insertion sites. Arrows indicate primer binding sites for RT-PCR analyses. (**B**) Transcript analysis of *PAT14* in wild type (WT), *pat14-1, pat14-2, PAT14g-GFP* in *pat14-2* (*PAT14g-GFP;pat14-2*) and *PAT14gC157S -GFP* in *pat14-2* (*mPAT14g-GFP;pat14-2*). *ACT2, ACTIN2*, was used as the internal control. (**C**) A representative plant at 40 days after germination (DAG) under long day (LD) condition from the corresponding genetic backgrounds shown in (**B**). (**D,E**) Electrolyte leakage (**D**) and relative chlorophyll contents (**E**) of wild type and *pat14-2* at W3, W4, and W5 were measured by using the 4^th^ pair of true leaves. Results are given as means ± standard deviation (SD), N = 30. * and *** indicate significant difference (Students’ *t*-test, P < 0.05 or P < 0.001 respectively). (**F**) Onset and progression of leaf senescence in LD-grown wild type and *pat14-2*. Green bars indicate days from leaf emergence to visible yellowing at the leaf tip (onset) while yellow bars indicate the time period (days) it takes from the first visible yellowing at the leaf tip to the leaf petiole (progression). The 4^th^ pair of true leaves was chosen for this measurement. Results shown are given as means ± SD, N = 30. (**G,H**) Number of rosette leaves at floral transition (**G**) and number of days to bolting (**H**). Data were collected from 30 LD-grown plants for each genetic background. Results are given as means ± standard deviation (SD). No significant difference was detected (*t*-test, P > 0.05).

Using a reverse genetic approach, we isolated and characterized two T-DNA insertion alleles for *PAT14* (Fig. 1A), named as *pat14-1* (SALK_026159) and *pat14-2* (GABI-KAT153A10). Transcript analysis showed that both alleles were null mutants (Fig. 1B). The *pat14* mutants did not differ from wild type during vegetative growth under long day (LD) condition (Supplemental Fig. 1). However, at approximately 4 weeks after germination (4WAG) when both wild type and mutants bolted, *pat14* mutants showed rapid leaf chlorosis while wild type stayed green for a more extended period (Fig. 1C, Supplemental Fig. 1). A green fluorescence protein (GFP) translational fusion of the *PAT14* genomic fragment driven by its own promoter (*PAT14g-GFP*) fully restored the mutant phenotype (Fig. 1C). Because *pat14-2* is a GABI-KAT insertion line that allows easier selection on plates, it was used for further analyses unless noted otherwise.

We carried out detailed analyses comparing *pat14-2* with wild type on several physiological parameters reflecting the senescing status of plants, such as ion leakage and chlorophyll contents^8^,^10^,^12^,^24^. The 4^th^ pair of true leaves was chosen for these analyses because of their close association with developmental programs as reported by others^6^,^16^,^19^. Ion leakage slightly increased from 3-week-old (W3) to 5-week-old (W5) in wild-type plants (Fig. 1D). By contrast, ion leakage of *pat14-2* leaves increased substantially over time, especially from W4, after plants have bolted and was significantly higher than wild type at W4 and W5 but not before (Fig. 1D). Consistent with the fact that wild type stayed green for an extended period after floral transition (Supplemental Fig. 1), contents of chlorophyll did not decrease from W3 to W5 in wild type (Fig. 1E). In stark contrast, chlorophyll levels began to drop from W4 in *pat14-2* (Fig. 1E). Accumulation of reactive oxygen species (ROS) and occurrence of cell death were increased in *pat14-2* (Supplemental Fig. 2).

Both the onset and progression of senescence were earlier in *pat14-2* than in wild type (Fig. 1F). To exclude the possibility that early senescence of *pat14-2* was due to altered developmental programs such as early flowering^2^,^4^, we analyzed flowering time based on the number of days to bolting and the number of rosette leaves upon floral transition because the two parameters faithfully reflect the developmental timing. No significant difference was detected between *pat14-2* and wild type either on the days to bolting or the number of rosette leaves (Fig. 1G,H). Because *pat14* mutants showed accelerated leaf senescence under LD condition only after floral transition, we wondered whether its function was induced by floral induction or just age related. To distinguish the two possibilities, we grew wild type, *pat14-2*, and *PAT14g-GFP;pat14-2* plants under short day (SD) condition. Two-month-old *pat14-2* showed accelerated leaf chlorosis than wild type or *PAT14g-GFP;pat14-2* plants under SD condition when phase transition was yet to occur (Supplemental Fig. 3). These results indicated that functional loss of *PAT14* specifically promoted senescence in an age-dependent manner without affecting the developmental timing.

### Functional loss of *PAT14* causes transcriptomic changes indicative of precocious leaf senescence

To provide more evidence that *PAT14* functional loss resulted in early senescence and also to gain insights into the molecular basis of the underlying mechanisms, we performed microarray analyses to compare transcriptomic changes during natural senescence versus by *PAT14* loss-of-function. RNAs were extracted from the 4^th^ pair of leaves from wild type (WT) or *pat14-2* (MU) at 21 DAG (W3) or 35 DAG (W5). Because flowering occurs around W4 (Supplemental Fig. 1), the 4^th^ pair of true leaves from W3 and W5 represent sink and source tissues respectively, two opposite statuses regarding nutrient remobilization^4^. In addition, qPCRs showed that several senescence associated genes, such as *SAG13* and *SAG21*, were hardly detectable at W3 but high in W5 in wild-type leaves (Supplemental Fig. 4), indicating a significant difference between the two time-points regarding natural senescence.

**Figure 2:**
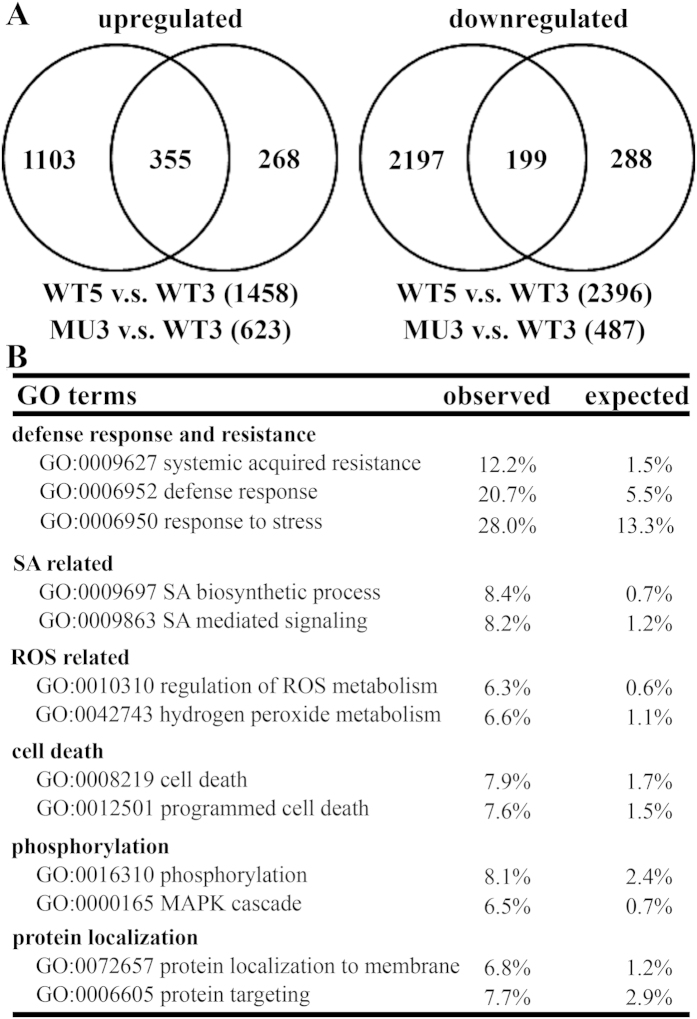
Functional loss of *PAT14* caused transcriptomic changes indicative of precocious leaf senescence. (**A**) Venn diagrams to illustrate the number of genes significantly upregulated or downregulated in wild type during development (WT5 v.s WT3) or by *PAT14* loss-of-function (MU3-WT3). The groups only include genes that show at least twofold upregulation or downregulation in the relevant experiments. Detailed gene lists are included in Dataset S1. (**B**) Functional categories overrepresented in the 623 upregulated genes by *PAT14* loss-of-function (MU3 v.s. WT3) according to GO Term analysis. All categories shown are significantly overrepresented (Student’s *t*-test, P < 0.01).

Arabidopsis Affymetrix GeneChip arrays were probed. In WT3, MU3, or WT5 samples, approximately 60% of the 24000 genes on the GeneChip showed detectable expression. Because *pat14-2* was significantly senesced at 5 WAG, which could have an overall impact on transcriptomes due to cellular dismantling, we did not include MU5 in the experiment. Using a twofold change of transcript abundance as the cutoff, 1458 or 2396 genes were upregulated or downregulated in WT5 v.s. in WT3 while only 623 or 487 genes were upregulated or downregulated in MU3 v.s. in WT3 (Fig. 2A and Supplemental Dataset 1). Because transcriptomic changes in WT5 v.s in WT3 encompass both senescence-related and developmentally regulated transcriptional changes while mutations at *PAT14* did not affect developmental programs, it was not a surprise that a much larger number of genes were transcriptionally changed in WT5 v.s. WT3 than in MU3 v.s. WT3 (Fig. 2A). A large percentage of genes showing different transcript abundance by *PAT14* loss-of-function (MU3 v.s. WT3) fall into natural senescence-induced and developmentally regulated transcriptomic changes in wild type (WT5 v.s. WT3) (Fig. 2A and Dataset S1). We verified results from microarray analyses by using qPCRs to test transcript abundance of representative genes whose upregulation or downregulation reflects senescence status (Fig. S4). Few Gene Ontology (GO) terms were over-represented among genes downregulated by *PAT14* loss-of-function (MU3 v.s. WT3), within which most related to organ morphogenesis and development (Fig. 2A, Supplemental Dataset 1). By contrast, GO terms including defense response, cell death, hormone signaling, transport, intracellular trafficking, ROS signaling, responses to stresses, and cellular signaling are over-represented among genes upregulated by *PAT14* loss-of-function (Fig. 2B and Supplemental Dataset 2). Among hormone-related functional categories, genes involved in SA biosynthesis and signaling were over-represented (Fig. 2B), indicating enhanced SA pathways by *PAT14* loss-of-function.

### *PAT14* suppresses age-dependent leaf senescence by repressing SA biosynthesis and signaling

**Figure 3:**
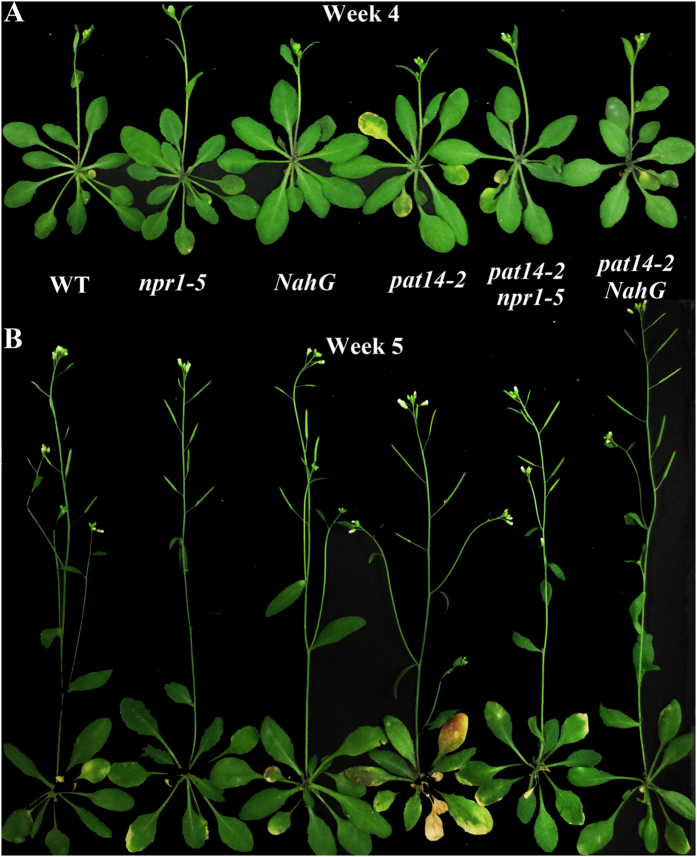
Mutations at SA biosynthesis and signaling suppressed age-dependent precocious leaf senescence by *PAT14* loss-of-function. (**A,B**) Representative images of wild type (WT), *npr1-5, NahG, pat14-2, pat14-2 npr1-5*, and *pat14-2 NahG* at 28 DAG (Week 4, A) or 35 DAG (Week 5, B).

The over-representation of SA biosynthetic and signaling pathways in *pat14-2* before the onset of senescence suggested that precocious leaf senescence during development by *PAT14* loss-of-function depended on SA biosynthesis and signaling. To test this hypothesis, we crossed *npr1-5*, a null mutant of *NONEXPRESSER OF PATHOGEN RELATED GENE1* (*NPR1*) impaired in most SA responses^11^,^25^, and *NahG* that encodes an SA hydroxylase to deplete endogenous SA^26^, with *pat14-2*. Under green house conditions, the SA deficient mutant *npr1-5* and *NahG* were undistinguishable from wild type (Fig. 3). However, introducing *npr1-5* or *NahG* into *pat14-2* substantially delayed the precocious leaf senescence either at 4 WAG (Fig. 3A) or 5 WAG (Fig. 3B), suggesting that precocious leaf senescence of *pat14-2* relies on intact SA biosynthesis and signaling.

**Figure 4:**
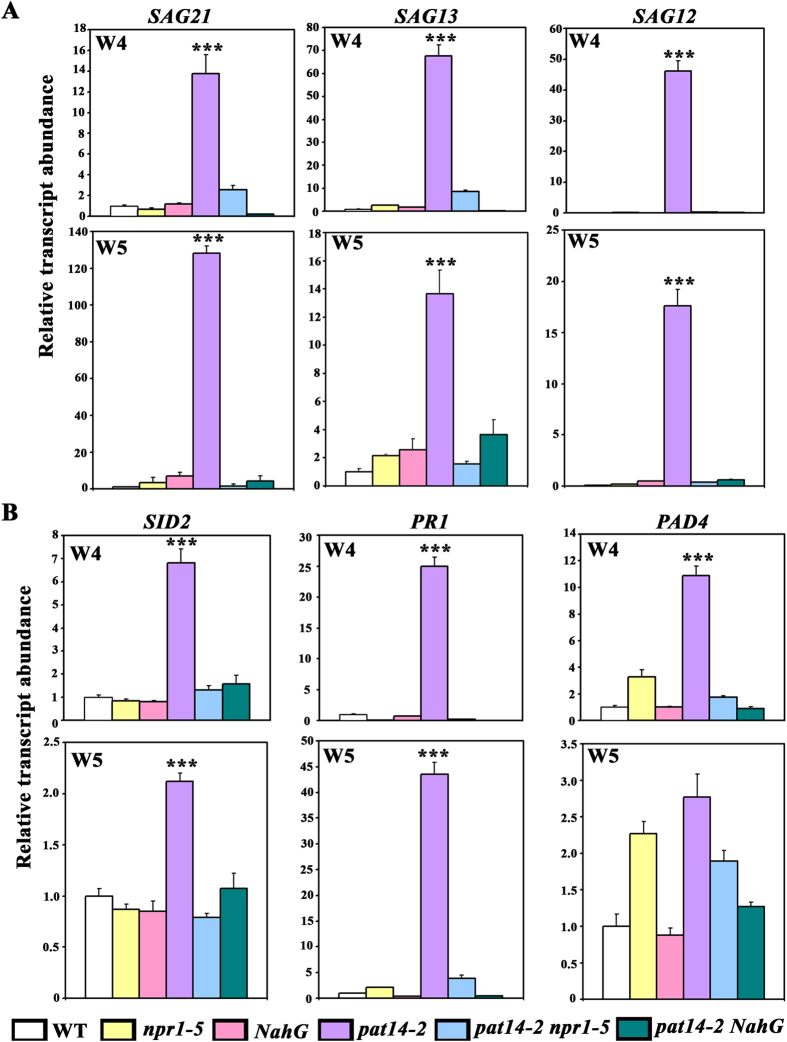
Mutations at SA biosynthesis and signaling suppressed age-dependent precocious leaf senescence by *PAT14* loss-of-function. (**A,B**) Relative transcript abundance of *SAG*s (*SAG12, SAG13* and *SAG21*) as well as SA responsive genes (*PAD4, PR1*, and *SID2*) in wild type, *npr1-5, NahG, pat14-2, pat14-2 npr1-5*, and *pat14-2 NahG* at week 4 (W4, **A**) or week 5 (W5, **B**) by qRT-PCRs. The 4^th^ pair of true leaves was used for RNA extractions. Results are given as means ± SD of one out of three independent experiments. Asterisks indicate significant difference from either of the other samples (*t*-test, P < 0.01).

To determine the senescence status of these mutants molecularly, we analyzed the expression of genes encoding senescence associated proteins and SA signaling proteins at 4 WAG and 5 WAG by quantitative real time PCRs. Consistent with its precocious leaf senescence, the *pat14-2* plants showed significantly higher expression of *SAG*s, such as *SAG12, SAG13* and *SAG21*, which were suppressed by the introduced *npr1-5* or *NahG* (Fig. 4A). Genes responding to SA signaling, such as *PAD4, PR1*, and *SID2*, were significantly induced by *PAT14* loss-of-function (Fig. 4B). Similar to that of *SAG*s, their induction in *pat14-2* was also suppressed by *npr1-5* or *NahG* (Fig. 4B). These results provided molecular evidence supporting the SA-dependent effect of *PAT14* on age-dependent leaf senescence.

### Overexpressing *PAT14* suppresses SA biosynthesis and signaling but is not sufficient to extend leaf longevity

**Figure 5:**
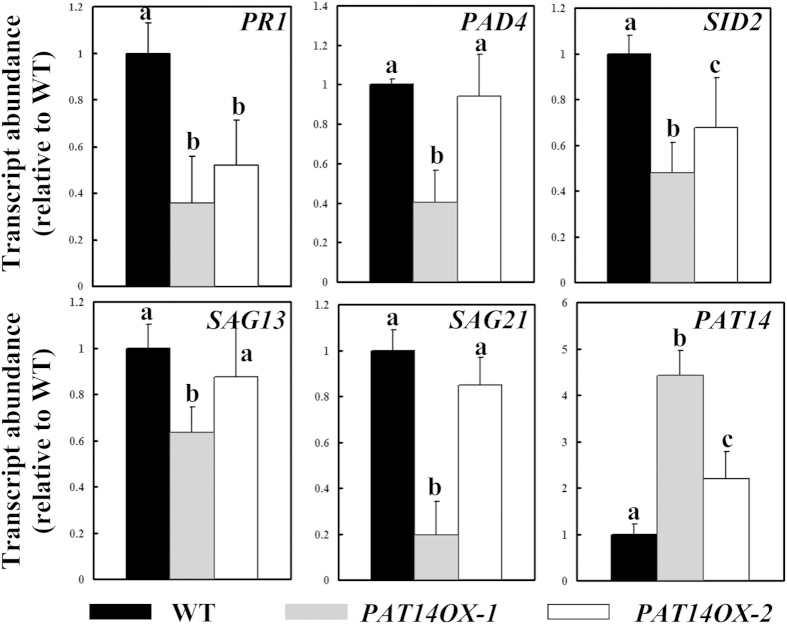
Overexpression of *PAT14* suppressed the expression of SA biosynthetic and signaling genes. Relative transcript abundance of SA responsive genes (*PAD4, SID2*, and *PR1*), two *SAG*s (*SAG13* and *SAG21*), and *PAT14* in wild type and two lines of *PAT14OX* at week 3 by qRT-PCRs. The 4^th^ pair of true leaves was used for RNA extractions. Results are given as means ± SEM, N = 3. Means with different letters are significantly different (*t*-test, P < 0.01).

To further verify that *PAT14* suppressed SA biosynthesis and signaling and to determine whether PAT14 gain-of-function had an opposite phenotype to that of mutants, i.e. extended leaf longevity, we generated a construct overexpressing *PAT14* using the *Pro*_*35S*_ promoter. More than twenty *Pro*_*35S*_:*PAT14* transgenic lines were obtained. However, no substantial difference was observed at the onset and progress of natural senescence between wild type and *PAT14* overexpression lines (Supplemental Fig. 5), suggesting that at least *PAT14* overexpression alone could not extend leaf longevity under regular growth conditions. However, we found that overexpressing *PAT14* significantly suppressed the expression of SA biosynthetic and signaling genes, such as *PR1, PAD4*, and *SID2*, at an extent correlating with the transcriptional levels of *PAT14* in different transgenic lines (Fig. 5). These results indicated that PAT14 indeed suppresses SA biosynthesis and signaling, which alone, however, is not sufficient to extend leaf longevity during age-dependent leaf senescence.

### Precocious leaf senescence of *pat14-2* was not dependent on other hormonal signaling

**Figure 6:**
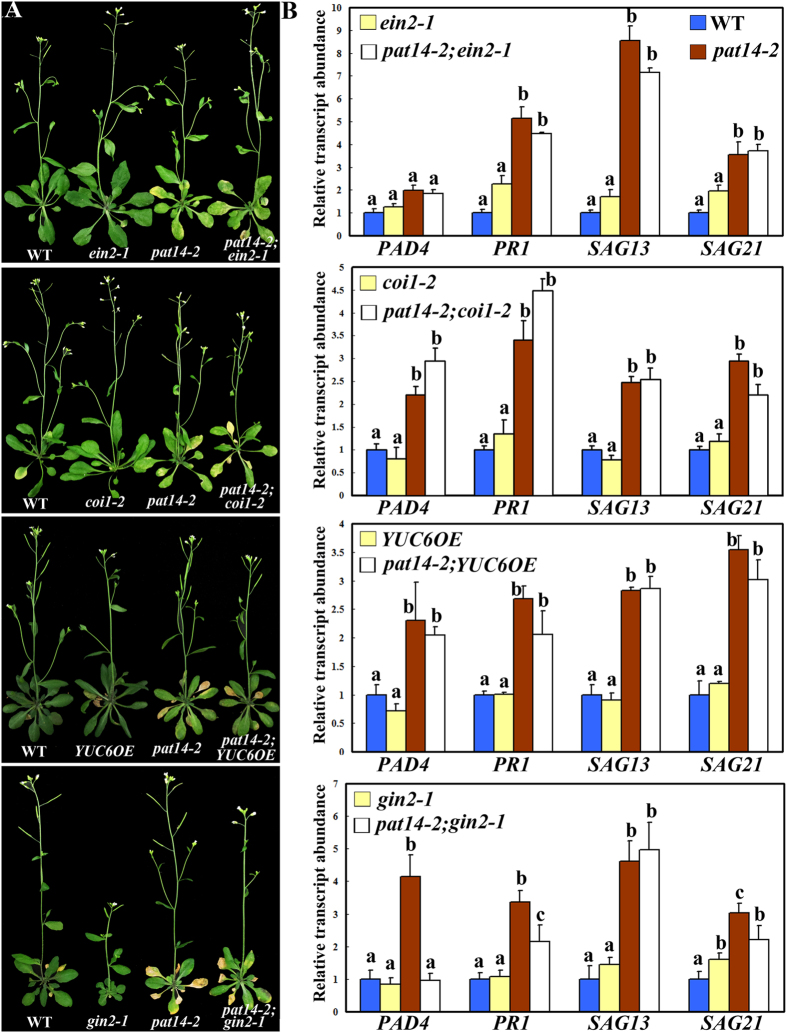
Disrupted ethylene or JA signaling as well as enhanced auxin or cytokinin signaling did not suppress the early senescence of *PAT14* loss-of-function. (**A**) Representative images of plants from the given genotypes at 5 weeks after germination (Week 5). (**B**) Relative transcript abundance of SA responsive genes (*PAD4* and *PR1*) and senescence-associated genes (*SAG13* and *SAG21*) in given genotypes at week 5 by qRT-PCRs. The 4^th^ pair of true leaves was used for RNA extraction. Results are given as means ± SEM, N = 3. Means with different letters for each genetic background are significantly different (*t*-test, P < 0.01).

Other hormones also play promoting or inhibiting roles in leaf senescence; the former including ethylene and JA while the latter including auxin and CK^1^,^2^. To determine whether *PAT14* controls leaf senescence also through other hormonal pathways, we introduced corresponding mutants or transgenic overexpression lines into *pat14-2* for their potential genetic interactions. Introducing *ein2-1*, an *ETHYLENE-INSENSITIVE2* (*EIN2*) mutant insensitive to ethylene^9^, or *coi1-2*, a *CORONATINE-INSENSITIVE1* (*COI1*) mutant insensitive to JA^27^ did not suppress the precocious leaf senescence of *pat14-2* (Fig. 6A,B). On the other hand, overexpressing *YUC6* to increase auxin biosynthesis^15^ or introducing *gin2-1* in which cytokinin signaling was enhanced^28^ did not suppress the precocious leaf senescence of *pat14-2* (Fig. 6A,B). These results indicated that *PAT14* regulates age-dependent leaf longevity specifically through the SA pathway.

### *PAT14* is not involved in carbon starvation-induced leaf senescence

Because leaf senescence is also induced by carbon starvation, we wondered whether *PAT14* was involved in carbon starvation-induced leaf senescence. To test that, we placed wild-type and *pat14-2* plants of 3 WAG in the dark for a certain period. Wild-type and *pat14-2* plants growing under LD condition in nutrient rich soil were comparable at 3 WAG (Supplemental Fig. 6). Dark treatment for 3 days (D3 - C) resulted in leaf chlorosis comparably between *pat14-2* and wild type (Supplemental Fig. 6). No substantial differences regarding leaf chlorosis were observed for *PAT14* overexpressing lines or the SA deficient mutant *npr1-5* or *NahG* (Supplemental Fig. 6). The result suggested that PAT14 is not involved in leaf senescence induced by carbon starvation.

### *PAT14* is constitutively expressed

**Figure 7:**
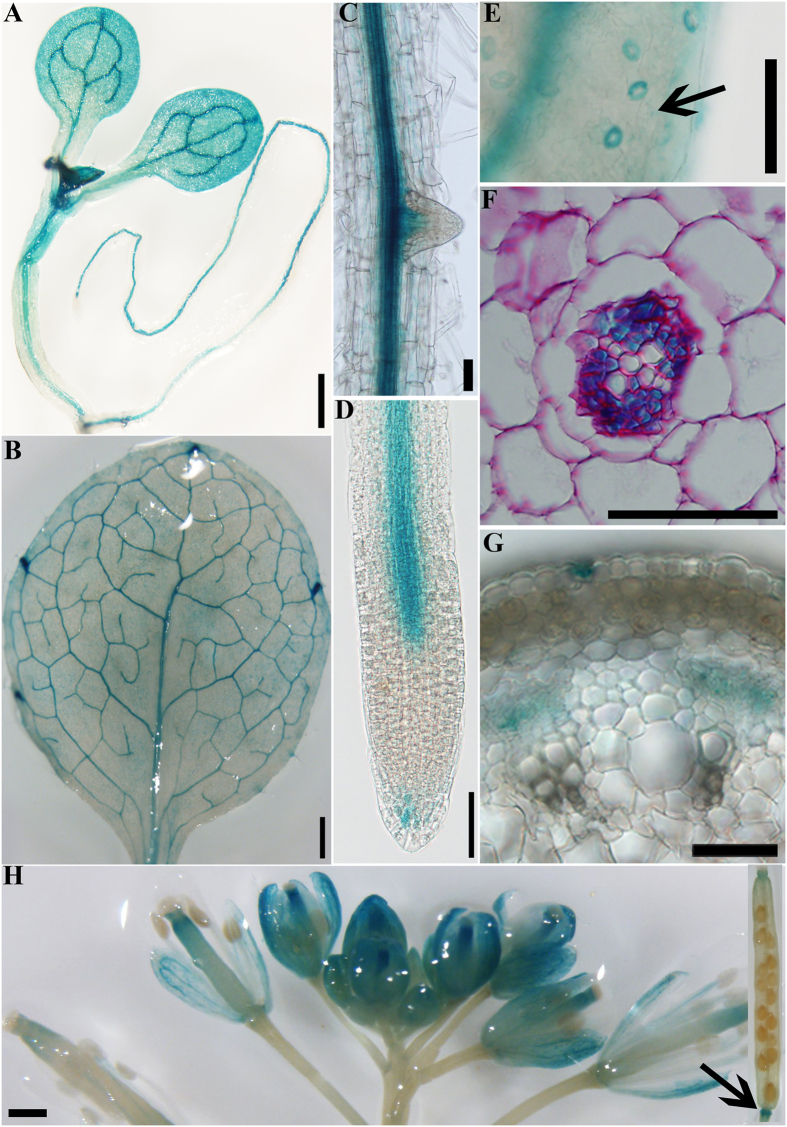
Expression of *PAT14* by histochemical analyses of *PAT14g-*GUS; *pat14-2* transgenic lines. (**A–H**) GUS signals are detected in seedlings (**A**), leaves especially veins (**B**), root vascular tissues (**C,D**) and lateral root primordial (**C**), guard cells (**E**), phloem tissues in roots (**F**) and stems (**G**), inflorescence (**H**) and floral organ abscission zone (H arrow). In total, 26 individual transgenic lines were analyzed and representative expression patterns are shown. Bars = 500 μm for (**A,B,E,H**); 50 μm for (**C,D,F**,**G**).

Because senescence associates with tremendous transcriptional changes^16^,^17^,^18^,^19^, the specific phenotype of *pat14* mutants prompted us to test whether *PAT14* was transcriptionally regulated during senescence. We generated a *PAT14g-GUS* that fully restored leaf longevity when introduced in *pat14-2* as did *PAT14g-GFP* (Fig. 1). By histochemical analysis of *PAT14g-GUS*;*pat14-2*, we found that *PAT14* was constitutively expressed in various tissues, such as seedlings (Fig. 7A), leaves (Fig. 7B), lateral and primary roots (Fig. 7C,D), stems (Fig. 7G), and inflorescences (Fig. 7H). Expression of *PAT14* was also detected in specific cell types, such as guard cells (Fig. 7E). *PAT14* was expressed most prominently in vascular tissues, specifically in the phloem (Fig. 7F,G).

We tested whether *PAT14* was regulated by senescence during development, by carbon starvation, or by SA through analyzing GUS signals of *PAT14g-GUS;pat14-2* transgenic plants of different ages, upon dark treatment, or upon treatment with an SA agonist, benzo (1,2,3) thiadizaole-7-carbothioic acid (BTH), respectively. GUS signals were not detectably different in leaves from W3 to W5 (Supplemental Fig. 7). Indeed, no senescence-associated transcriptional change was identified for *PAT14* based on microarray studies of natural senescence^16^. Histochemical analysis on *PAT14g-GUS;pat14-2* seedlings did not show signal enhancement upon dark treatment or by BTH treatment (Supplemental Fig. 7). To confirm the constitutive expression of *PAT14*, we performed quantitative real-time PCRs (qPCRs) on wild-type plants at W3, W4, and W5. Results obtained by qPCRs were consistent with the GUS data such that no significant difference was detected during development for the expression of *PAT14* (Supplemental Fig. 7), in contrast with many senescence-associated genes (*SAG*s) whose expression increased significantly when plants age (Supplemental Fig. 4). Because *PAT14* is constitutively expressed, its roles in leaf senescence during plants aging could be regulated at the post-transcriptional level such as by post-translational modifications.

### PAT14 is localized at the Golgi, the *trans*-Golgi network/early endosome, and prevacuolar compartment

**Figure 8:**
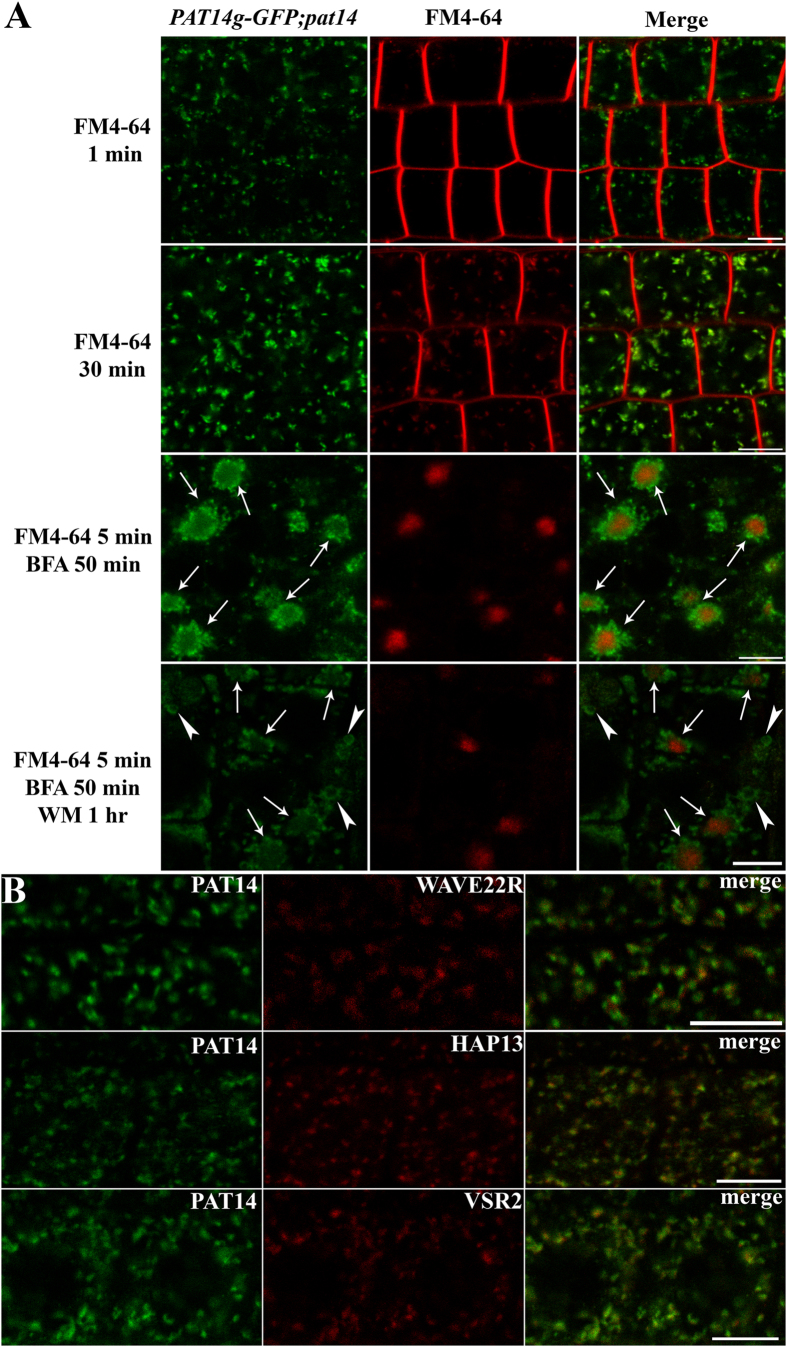
Arabidopsis PAT14 is localized at the Golgi, the TGN/EE, and PVC/MVB. (**A**) Confocal fluorescence imaging of *PAT14g-GFP* in *pat14-2* (*PAT14g-GFP;pat14-2*) stained with FM4-64. Arrows indicate BFA compartments in which both FM4-64 and PAT14-positive TGN/EE accumulates in the center surrounded by PAT14-positive Golgi stacks. Arrowheads indicate ring-shaped PVC/MVB labeled by PAT14 upon wortmannin treatment. (**B**) *PAT14g-GFP* was introduced in transgenic plants expressing RFP-fused markers for Golgi (WAVE22R), TGN/EE (HAP13), and PVC/MVB (VSR2). Partial co-localization was observed for all combinations. Bars = 7.5 μm for (**A**); 5 μm for (**B**).

PATs are multi-span transmembrane proteins whose subcellular localization plays a key role in their functional specificity^21^,^22^. The *PAT14g-GFP* transgene fully restored leaf longevity after floral transition in *pat14-2* (Fig. 1C), indicating that the GFP fusion did not disturb its function and thus would reflect its native subcellular localization. In *PAT14g-GFP*;*pat14-2*, GFP signals were present in vesicular structures (Fig. 8A). To determine the identity of these vesicles, we applied fluorescence colabeling by using the lipophilic fluorescence dye FM4-64 that is internalized to several endomembrane compartments through endocytic trafficking^29^. FM4-64 was detected at punctuate vesicles as early as 5 min after pulse labeling (Fig. 8A), which represent the *trans*-Golgi network/early endosome (TGN/EE). A portion of GFP signals overlapped with the internalized FM4-64 signals (Fig. 8A), indicating its localization at the TGN/EE. We then applied the fungal toxin Brefeldin A (BFA) at the presence of cycloheximide (CHX). BFA interferes with post-Golgi trafficking, and thus results in membrane aggregates called the “BFA compartments” that contain TGN/EE cores and surrounding Golgi stacks^30^ while CHX inhibits *de novo* protein synthesis. BFA treatment caused FM4-64-positive signals to form so-called BFA compartments (Fig. 8A). Under such treatment, a subset of PAT14-positive signals colocalized with FM4-64 in the “core” of BFA compartments while another subset surrounded the FM4-64-core (Fig. 8A), indicating that PAT14 is present both at the Golgi and the TGN/EE. Beside its localization at the Golgi and the TGN/EE, a portion of GFP signals was insensitive to even prolonged BFA treatment (Fig. 8A). To determine the identity of these vesicles, we applied Wortmannin (WM) after BFA treatment. WM is mostly noted for its inhibitory role on vacuolar fusion of prevacuolar compartments/multivesicular bodies (PVC/MVB) and thus resulted in ring-shaped vesicles of PVC/MVB identity^31^. Application of WM resulted in the fusion of the remaining punctate vesicles into ring-shaped compartments unassociated with the BFA-induced aggregates (Fig. 8A), indicative of PVC/MVB. Thus, we concluded that PAT14 is localized at several endomembrane compartments including the Golgi, the TGN/EE, and the PVC/MVB.

To gain more evidence for the subcellular localization of PAT14, we also introduced *PAT14g-GFP* into various transgenic lines expressing RFP-fused subcellular markers, including the Golgi marker WAVE22R^32^, the TGN/EE marker HAP13-RFP^33^ and the PVC/MVB marker VSR2-RFP^34^. Partial co-localization of PAT14 with all three markers again demonstrated its localization at these endomembrane compartments (Fig. 8B).

Compared to the fully restored leaf longevity of *pat14-2* by *PAT14g-GFP*, a mutation that disrupted the key catalytic Cys within its DHHC motif (PAT14gC157S) thus potentially abolished its palmitoylation activity^35^,^36^ failed to rescue the early senescence phenotype of *pat14-2* (Fig. 1C). This result suggested that the function of PAT14 during senescence was due to palmitoylation of its substrate(s). However, it was also possible that the point mutation altered its subcellular localization or stability, resulting in the failure of complementation. To exclude this possibility, we used confocal fluorescence microscopy to image the localization of *PAT14gC157S-GFP*;*pat14-2* by the same fluorescence colabeling and pharmacological treatments. Our results clearly showed that the C157S point-mutation did not interfere with the native localization or stability of PAT14 (Supplemental Fig. 8). Thus, we concluded that PAT14 promotes leaf longevity through its substrate palmitoylation.

### Evolutionarily conserved function of PAT14

PATs are encoded by multi-gene families in all eukaryotes, including plants. PATs from a given plant species are quite diverse in sequences outside their catalytic DHHC motifs, implying neo-functionalization during evolution after genome duplications^23^,^37^,^38^. However, proteins sharing high sequence identity with PAT14 were identified from various plant species ranging from the unicellular Chlamydomonas to various higher plant species (Supplemental Fig. 9), suggesting that functional diversification of *PAT14* occurred early and was retained during evolution.

**Figure 9:**
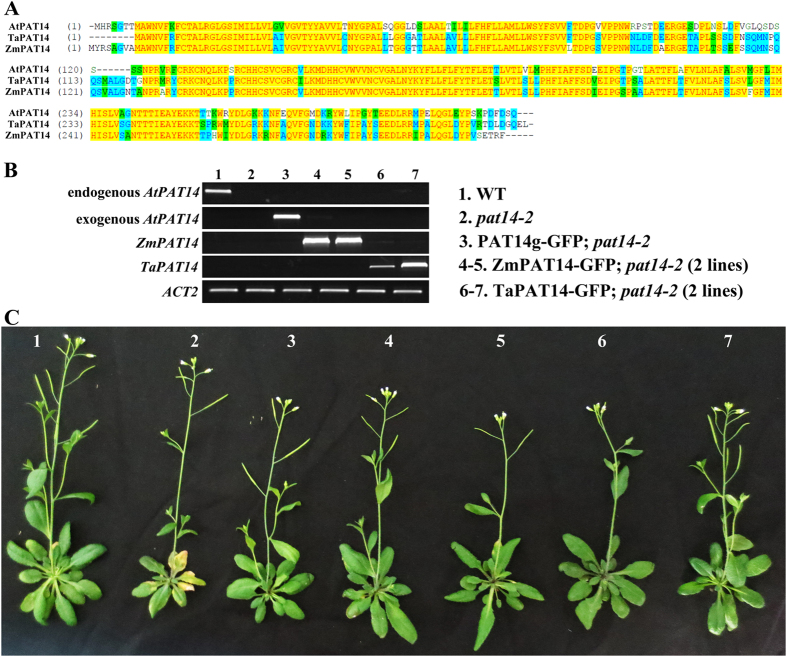
***P**AT14* homologs from maize and wheat suppressed the precocious leaf senescence of pat14-2. (**A**) Sequence alignment of Arabidopsis PAT14 and its homologs from maize (ZmPAT14) and wheat (TaPAT14). Yellow residues are consensus residues while blue or green residues represent residues replaced with other residues of similar characteristics. Sequence alignment was performed with Vector NTI (Invitrogen). UNIPROT accession number for ZmPAT14 is CPOBY3 and for TaPAT14 is W5B4H0. (**B**) Transcript analysis of Zm*PAT14* or *TaPAT14* in *pat14-2. ACT2* was used as the internal control. (**C**) Representative complemented plants at reproductive stage corresponding to the genotypes showing in (**B**).

To determine whether the function of *PAT14* was conserved during evolution, we isolated PAT14 homologs from two major crops, maize and wheat, for which proper timing of leaf senescence is crucial for increasing seed yield. Sequence alignment showed that the central hydrophilic loop containing the DHHC motif of PAT14s are highly conserved (Fig. 9A, Supplemental Fig. 9), presumably important for the recognition of specific cargos. In addition to the catalytic domain, both the C-terminal cytoplasmic tails and the N-terminal hydrophilic region are also highly conserved among PAT14 homologs (Fig. 9A, Supplemental Fig. 9), suggesting key regulatory function by non-catalytic domains. We thus generated constructs containing *Pro*_*35S*_-driven GFP-fused ZmPAT14 and TaPAT14 and introduced the transgenes into *pat14-2* (Fig. 9B). The fact that the expression of *ZmPAT14-GFP* and *TaPAT14-GFP* fully restored leaf longevity of *pat14-2* (Fig. 9C,D) indicates functional conservation.

## Discussion

We show in this study the identification and characterization of a novel component involved in leaf senescence during development. The *pat14* mutants behaved similarly to wild type until floral induction when accelerated cell death occurred in the mutants (Fig. 1, Supplemental Figs 1 and 2). However, the function of *PAT14* during leaf senescence is not dependent on floral induction because *pat14* under SD condition also senesced earlier before floral transition was yet to occur (Supplemental Fig. 3). In addition, functional loss of *PAT14* did not alter the sensitivity toward artificial carbon starvation (Supplemental Fig. 6), indicating its specific involvement in age-dependent processes.

Transcriptomic analysis revealed the possible involvement of SA signaling in *PAT14* loss-of-function (Fig. 2). This was confirmed molecularly by analyzing the relative expression of SA responsive genes, which showed elevated expression in *pat14-2* (Fig. 4, Supplemental Fig. 4) but reduced expression by *PAT14* overexpression (Fig. 5). The SA-dependency was further verified by genetic analysis. Introducing *NahG* to reduce SA accumulation suppressed the leaf chlorosis and elevated expression of *SAG*s of *pat14-2* during development (Figs 3 and 4). Although it was shown that some SA-inducible genes were not dependent on NPR1 and thus suggesting other components downstream of SA^11^, we showed genetically and molecularly that *npr1-5* fully suppressed the precocious leaf senescence of *pat14-2* (Figs 3 and 4), suggesting that *PAT14* mediates SA signaling in an NPR1-dependent way. On the other hand, manipulating other hormonal signals by using corresponding mutants or overexpressors did not affect the precocious leaf senescence of *pat14-2* (Fig. 6). Previous transcriptomic and genetic studies suggested that SA signaling is specifically involved in natural but not starvation-induced leaf senescence^11^,^17^. Thus, the NPR1-dependent SA signaling explains well the phenotype of *PAT14* loss-of-function.

Unlike most genes involved in senescence, *PAT14* is not transcriptionally induced during leaf senescence or by carbon starvation (Supplemental Fig. 7). In fact, although overexpressing *PAT14* did suppress the expression of SA responsive genes, it did not significantly promote leaf longevity by itself (Supplemental Fig. 5). Both results indicate that PAT14 is regulated post-transcriptionally during senescence. However, despite its constitutive expression, *PAT14* is prominent in the phloem of root and aerial parts (Fig. 7). Precocious cell death in *pat14-2* leaves occurred mostly proximal to leaf veins rather than spreading all over the old leaves (Supplemental Fig. 2). Phloem plays a key role in nutrient remobilization from sources to sinks. Because nutrient remobilization is an important issue to be addressed during floral induction and carbon starvation when developing seeds or newly initiated leaves become new sinks, the failure to satisfy the need due to *PAT14* loss-of-function may be partially compensated by the accelerated cell death of old leaves. This is certainly an interesting possibility to be investigated in the future. In addition, SA migrates from the site of infection to other parts of plants through phloem, which is critical for systemic acquired resistance during pathogen infection^39^. Considering the enhanced SA signaling on site in *PAT14* loss-of-function and the enriched expression of *PAT14* in phloem, the intriguing possibility that *PAT14* facilitates long-distance SA transport is certainly worthy of future exploration.

The C157S mutation potentially disrupting the catalytic activity of PAT14^22^,^35^,^36^ abolished its ability to rescue *pat14-2* (Fig. 1), suggesting that substrate palmitoylation is responsible for the functionality of PAT14 during natural senescence. Currently, it is still a technical challenge to pinpoint substrates of a given PAT. A biotin switch isobaric tagging approach was developed, which theoretically can identify differentially palmitolyated proteins by comparing palmitoyl-proteomics of wild type and those of *PAT* mutants^40^,^41^. However, a recent attempt using this approach to identify substrates of Arabidopsis TIP GROWTH DEFECTIVE 1/PAT24 (TIP1)^35^ resulted in over a hundred proteins that are significantly under-palmitoylated by *TIP1* loss-of-function^41^. Even if the substrates of TIP1 are among the pool, it will be an unfathomable task to pinpoint them.

On the other hand, the localization of PAT14 will help to reveal the identity of its substrates. The localization of PAT14 (Fig. 8, Supplemental Fig. 8) indicates that it is involved in vacuolar trafficking. Earlier studies indicated that vacuolar trafficking may play a key role in chlorophyll degradation processes during senescence^42^. Recently, several studies showed that disrupting molecular components critical for vacuolar trafficking resulted in precocious leaf senescence. Functional loss of *AMSH1* and *VACUOLAR PROTEIN SORTING2.1* (*VPS2.1*), encoding proteins regulating vacuolar trafficking, resulted in early senescence and hypersensitivity to artificial carbon starvation^34^. Genetic interference of a few other genes encoding vacuolar trafficking regulators, such as VPS and Rab GTPases, also resulted in precocious leaf senescence^43^, ^44^,^45^,^46^. Indeed, microarray data indicated that vacuolar activities are among the most over-represented intracellular activities enhanced during natural senescence^16^. Thus, it is likely that PAT14 regulates the membrane association and activities of protein factors whose activity is important for vacuolar function specifically during senescence. Potential candidates are SNAREs, a large number of which are likely subjected to palmitoylation^21^,^22^,^41^ and critical for the specificity of membrane fusion^47^. Arabidopsis genome encodes a large number of SNAREs^47^ that are localized differentially at Golgi and post-Golgi compartments^48^. Cytological, genetic, as well as phenotypic analyses of SNAREs will help reveal the factors responsible for PAT14-mediated leaf senescence.

## Methods

### Plant materials, growth conditions, and histochemical analyses

The Arabidopsis T-DNA insertion lines, SALK_026159 (*pat14-1*) and GABI-KAT153A10 (*pat14-2*) were obtained from ABRC (http://www.Arabidopsis.org). *Arabidopsis* Columbia-0 ecotype was used as the wild type. Arabidopsis plants were grown as described^36^. Stable transgenic plants were selected on half-strength MS supplemented with 30 μg/ml Basta salts or 7.5 mg/ml sulfadiazine (Sigma, http://www.sigmaaldrich.com). The allelic mutant *pat14-2* was used to generate double mutants with *npr1-5, NahG, ein2-1, coi1-2, gin2-1*, and *YUC6* overexpression lines by crosses. GUS histochemical analyses were performed as described^36^. DAB staining for H_2_O_2_ and tryphan blue staining for cell death were performed as described^33^.

### PCR, RT-PCR, and qPCR

Mutants of *PAT14* were analyzed by genotyping PCR using the following primers: ZP1147/ZP1771 for *PAT14*, LB1/ZP1771 for *pat14-1*, ZP8/ZP1203 for *pat14-2.* For RT-PCR and quantitative real-time PCR analyses, total RNAs were extracted using the RNeasy Plant miniprep kit according to the manufacturer’s instructions (Qiagen). Reverse transcriptions were performed using Superscript^TM^ III Reverse Transcriptase with on-column DNase-I treatment (Invitrogen). The following primer pairs were used in RT-PCRs to characterize mutants or complemented mutants: F1/R1 for endogenous *PAT14*, ZP1599/ZP1600 for exogenous *PAT14* or *PAT14-C157S*, ZP1506/ZP11 for *ZmPAT14*, and ZP1628/ZP2682 for *TaPAT14*. Arabidopsis *ACTIN2* was used as the internal control for RT-PCRs^49^. The qPCR analyses were performed as described^36^. Primers in qRT-PCR analyses are as followed: ZP1581/ZP1582 for *SAG13*, ZP3139/ZP3140 for *SAG12*, ZP1498/ZP1499 for *SAG21*, ZP2190/ZP2191 for *PAD4*, ZP2319/ZP2320 for *SID2*, ZP2304/ZP2305 for *PDF1.2*, and ZP1583/ZP1584 for *PR1*. Primers are listed in Table S1.

### Plasmid construction

All constructs were generated using the Gateway^TM^ technology (Invitrogen). Entry vectors for the coding sequences of genes were generated in the pENTRY/SD/D-TOPO vector (Invitrogen). Primers for generating entry vectors containing corresponding coding sequences are as followed: ZP1399/ZP1400 for *YUC6*, ZP1506/ZP1509 for *ZmPAT14*, and ZP2681/ZP2682 for *TaPAT14*. The destination vector for *Pro*_*35S*_:*YUC6* and *Pro*_*35S*_:*PAT14-GFP* was described earlier^50^. The entry vector for the *PAT14* genomic sequence together with the 1305 base pair sequence upstream of its start codon (*PAT14g*) was generated by using the primer pair ZP1262/ZP1263. A C157S mutation was generated by site-directed mutagenesis from the *PAT14g* entry vector. The destination vector for *PAT14g-*GFP and *PAT14gC157S*-GFP translational fusion constructs was described earlier^36^. The destination vector for the *PAT14g*-GUS translation fusion construct was obtained from ABRC^51^. The destination vector for constitutively expressing ZmPAT14- and TaPAT14-GFP was described^50^. Expression vectors were generated by LR reactions using LR Clonase (Invitrogen). Primers are listed in Supplemental Table 1.

### Quantification of Chlorophyll contents and ion leakage

To measure the Chlorophyll contents and ion leakage, the 4^th^ pair of rosette leaves from 21 DAG (week 3), 28 DAG (week 4) or 35 DAG (week 5) plants were used. Chlorophyll was extracted and measured as described^24^. For ionic leakage measurement, the leaf discs without the major veins were collected and infiltrated in 10 ml deionized water with vacuum till the leaf discs were immersed in the water. Conductivity of the solution was measured after gentle agitation at root temperature for 1 hr. Total ionic strength was measured after the solution was heated in 100 °C water bath for 10 min and cooled to root temperature. The leaked ions were represented as the percentage of the initial conductivity versus the total conductivity.

### Pharmacological treatment and confocal microscopy

FM4-64 uptake, BFA treatments, and laser scanning confocal fluorescence imaging were performed as described^33^. For the combined BFA and WM treatment, 4 DAG seedlings were first pulse-labeled with 4 μM FM4-64 for 5 min. After three times of washing, seedlings were incubated in 1/2 MS supplemented with 50 μM BFA for 50 min. Seedlings were then incubated in fresh 1/2 MS supplemented with 50 μM BFA and 33 μM WM for 1 hr before being examined. BTH dissolved in water (100 μM) was sprayed on 1-week-old seedlings (for GUS histochemical analysis) or 3-week-old plants (for qPCRs). Materials were collected at designated time points (12 hrs–48 hrs) after spray for analysis.

### DNA microarray and data analyses

Three independently derived sets of wild-type (WT) or *pat14-2* (MU) plant materials either at W3 (WT3 or MU3) or at W5 (WT5 or MU5) were used for microarray analyses using the Affymetrix GeneChip® Arabidopsis ATH1 Genome Array, which was performed by Shanghai Biotechnology Co., Ltd. (www.ebioservice.com). Total RNAs were extracted using TRIZOL Reagent (Life technologies) following the manufacturer’s instructions and RNA integrity was inspected by an Agilent Bioanalyzer 2100 (Agilent technologies). Qualified total RNA was further purified by RNeasy micro kit (QIAGEN) and RNase-Free DNase Set (QIAGEN). Total RNAs were amplified, labeled and purified by using GeneChip 3’IVT Express Kit (Affymetrix) followed the manufacturer’s instructions to obtain biotin labeled cRNAs. Array hybridization and wash was performed using GeneChip® Hybridization, Wash and Stain Kit (Affymetrix) in Hybridization Oven 645 (Affymetrix) and Fluidics Station 450 (Affymetrix) followed the manufacturer’s instructions. Slides were scanned by GeneChip® Scanner 3000 (Affymetrix) and Command Console Software 3.1 (Affymetrix) with default settings. Raw data were normalized by MAS 5.0 algorithm, Gene Spring Software 11.0 (Agilent technologies). GO classification of genes exhibiting at least twofold changes in transcript levels was conducted using AmiGO online tool (version 1.8) with default settings^52^. Analyses were based on May 24^th^, 2014 release of GO database. Gene IDs and their putative functions were assigned using TAIR as the database filter. The expected ratio indicates the percentage of genes of the designed GO term within all genes represented on the GeneChip array.

## Additional Information

**Accession Numbers**: Sequence data from this article can be found in the GenBank databases under the following accession numbers: At3g60800 for PAT14, At5g25620Z for YUC6, At5g45900 for ATG7, At1g64280 for NPR1, At2g39940 for COI1, At4g29130 for GIN2, At2g29350 for SAG13, At5g45890 for SAG12, At4g02380 for SAG21, At3g52430 for PAD4, At1g74710 for SID2, At5g44420 for PDF1.2, and At2g14610 for PR1.

**How to cite this article**: Zhao, X.-Y. *et al.* Precocious leaf senescence by functional loss of *PROTEIN S-ACYL TRANSFERASE14* involves the NPR1-dependent salicylic acid signaling. *Sci. Rep.* **6**, 20309; doi: 10.1038/srep20309 (2016).

## Supplementary Material

Supplementary Information

Supplementary Dataset 1

Supplementary Dataset 2
