# Supplementary material for: Precocious leaf senescence by functional loss of PROTEIN S-ACYL TRANSFERASE14 involves the NPR1-dependent salicylic acid signaling
Source: Sci Rep. 2016 Feb 4;6:20309. doi: 10.1038/srep20309 (PMC4740857; doi:10.1038/srep20309)
Supplement: Supplementary Information [file srep20309-s1.pdf]

Supplemental Information for

Precocious leaf senescence by functional loss of *PROTEIN S-ACYL TRANSFERASE14* involves the NPR1-dependent salicylic acid signaling.

Xin-Ying Zhao[1], Jia-Gang Wang[1], Shi-Jian Song, Qun Wang, Hui Kang, Yan Zhang[*], Sha Li[*]

State Key Laboratory of Crop Biology, College of Life Sciences, Shandong Agricultural University, Tai'an, 271018, China

[1]These authors contributed equally to this work. [*] Corresponding author, email shali@sdau.edu.cn, yzhang@sdau.edu.cn

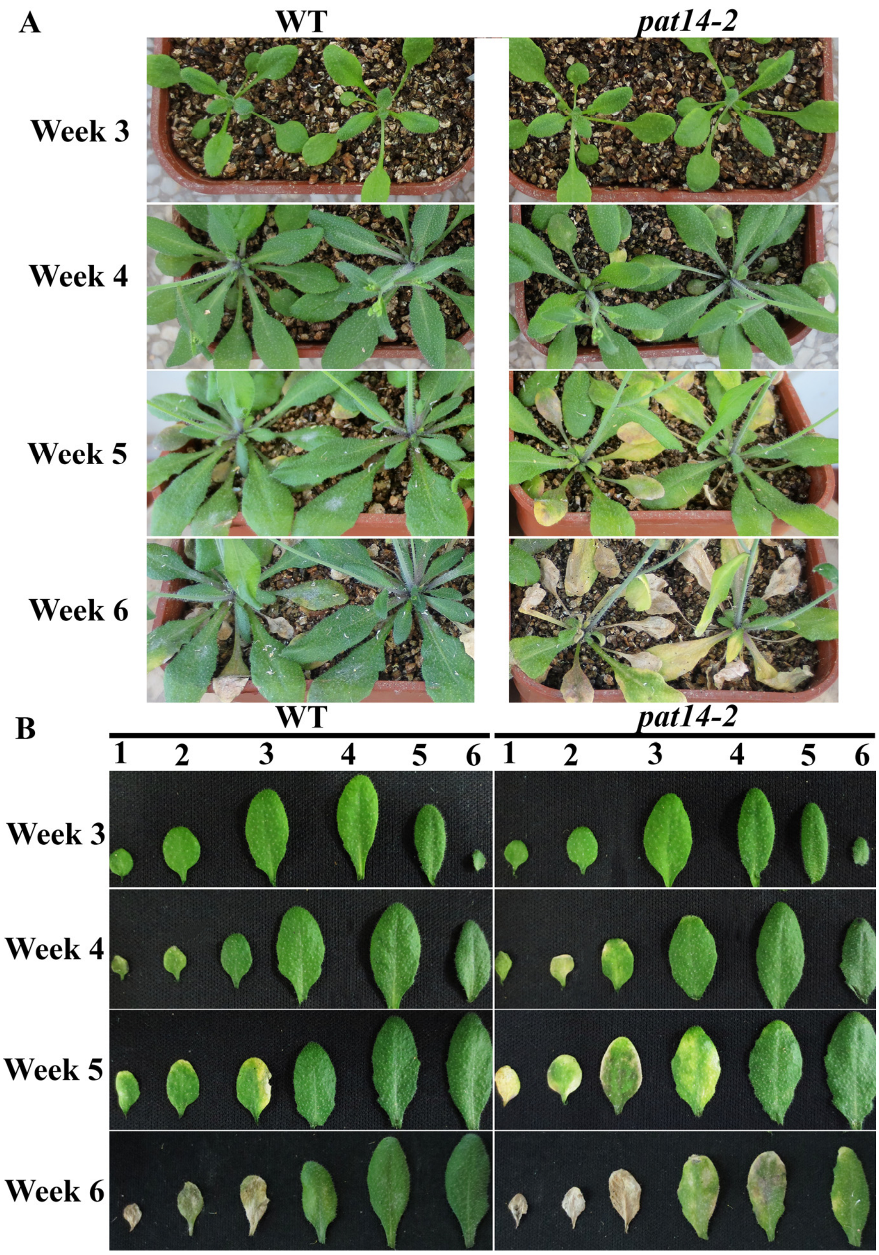

Supplemental Figure 1. Functional loss of *PAT14* resulted in early senescence. (A) Wild-type and *pat14-2* plants growing side-by-side under LD condition at 21 DAG (Week 3), 28 DAG (Week 4), 35 DAG (Week 5), or 42 DAG (Week 6). (B) Line-ups of leaves (one each from the 1st to the 6th pair of true leaves) detached from representative wild-type or *pat14-2* plants at indicated time points.

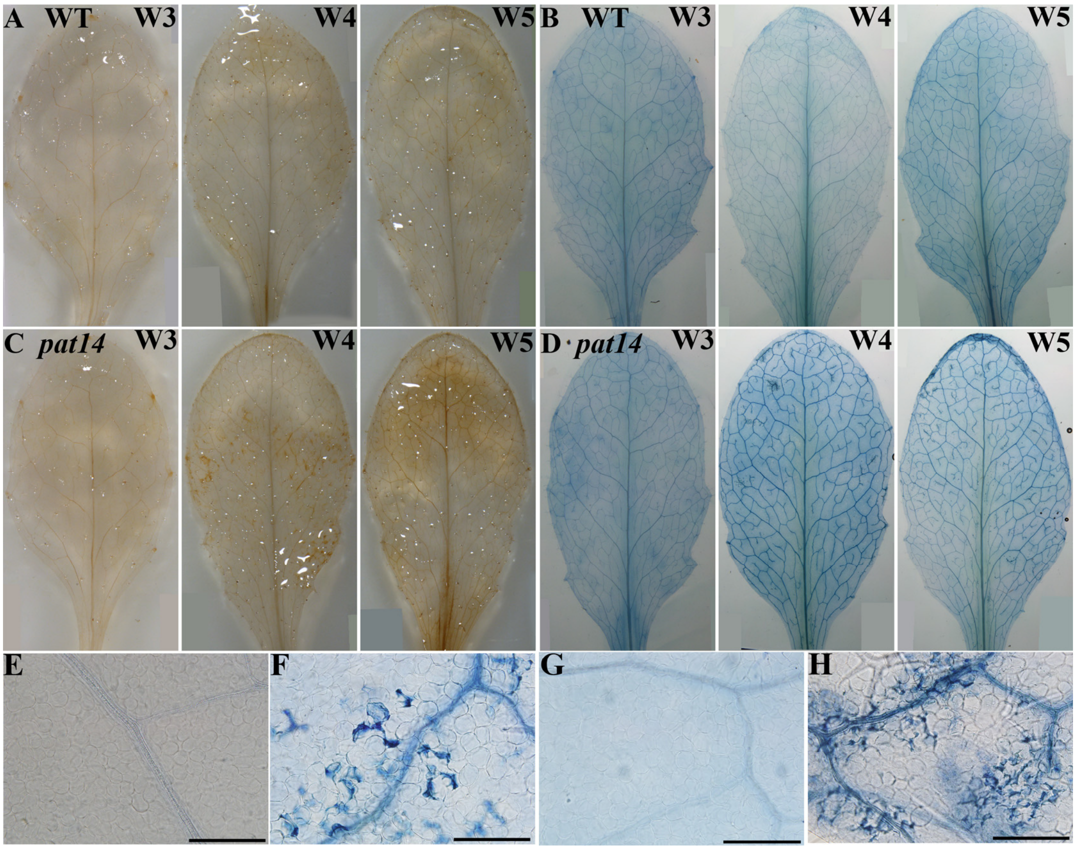

Supplemental Figure 2. Functional loss of *PAT14* resulted in enhanced ROS production and precocious cell death. DAB staining of the 4th pair of true leaf from wild type (A) or from *pat14-2* (C) is shown at indicated time points. Tryphan blue staining of the 4th pair of true leaf from wild type (B) or from *pat14-2* (D) is shown at indicated time points. Close-up images of tryphan blue-stained 4th pair of true leaf from wild type (E, G) or *pat14-2* (F, H) are collected at W4 (E, F) or at W5 (G, H). Bars = 200 µm for (E-H).

.

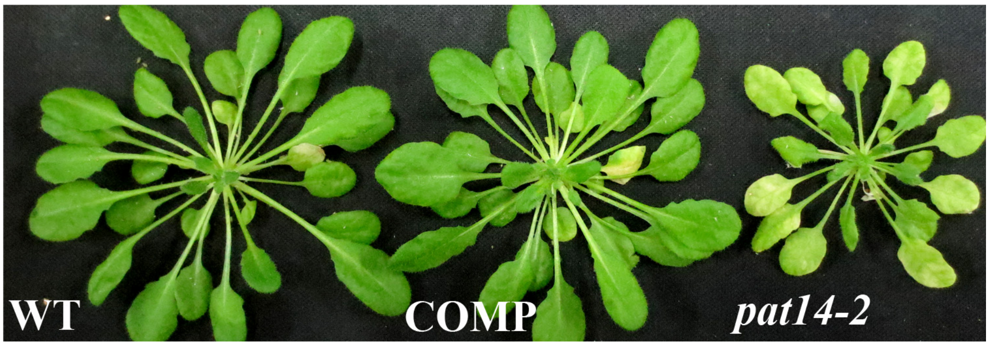

Supplemental Figure 3. Functional loss of *PAT14* caused precocious leaf senescence under short day (SD). A representative wild-type (WT), *PAT14g-GFP;pat14-2* (Comp) and *pat14-2* plant growing under SD condition for 2 months.

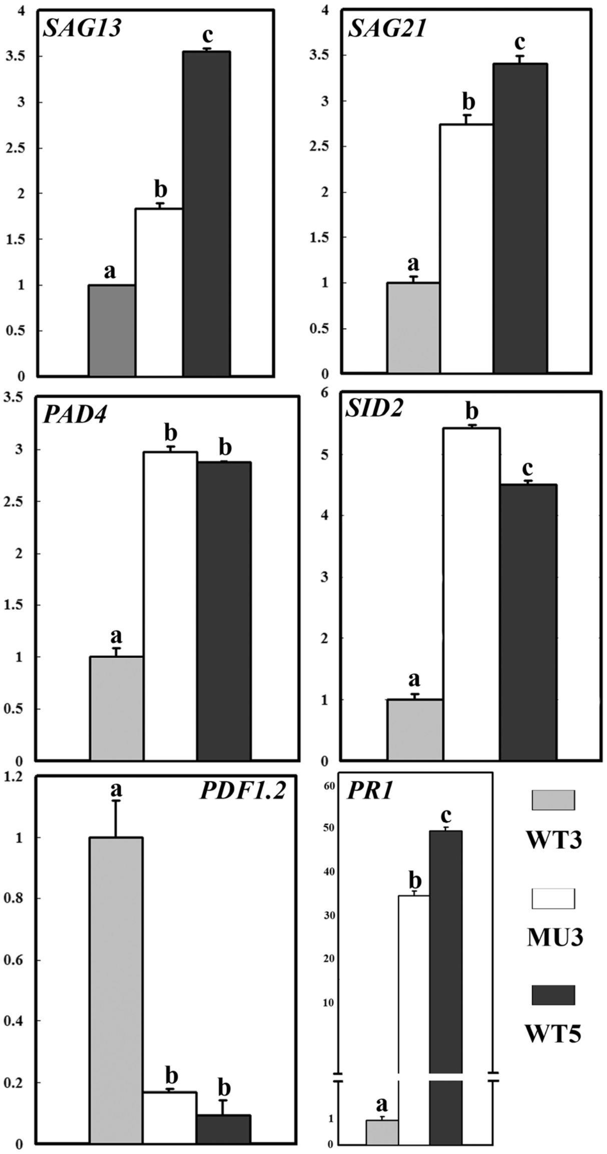

Supplemental Figure 4. Functional loss of *PAT14* caused transcriptional changes indicative of precocious leaf senescence. Quantitative real-time PCR analyses of genes showing comparable changes of transcript abundance both during natural senescence and by *PAT14* loss-of-function based on the microarray, including *SAG13*, *SAG21*, *PAD4*, *SID2*, *PDF1.2*, and *PR1*. Results are given as means ± SEM, N=3. Means with different letters for each gene are significantly different (*t*-test, P<0.01).

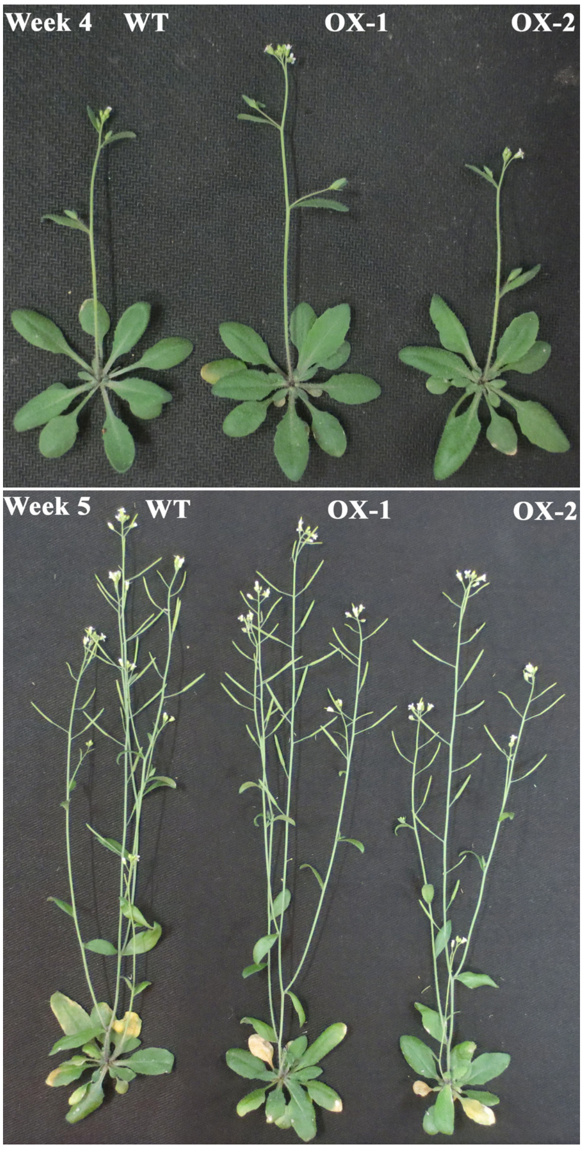

Supplemental Figure 5. Overexpression of *PAT14* did not delay age-dependent leaf senescence. WT and two representative lines of *PAT14OX* (OX-1 and OX-2) at 4 weeks or 5 weeks after germination (Week 4 or Week 5) under long day condition.

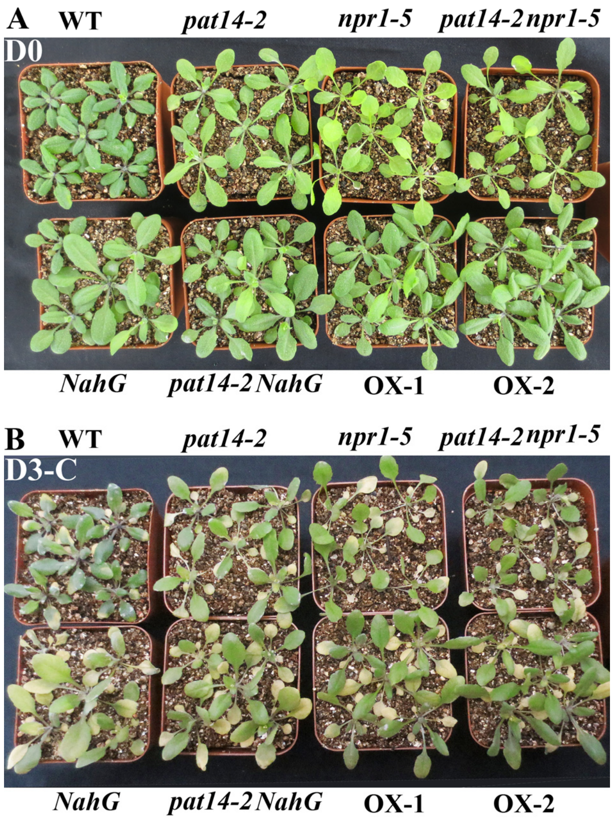

Supplemental Figure 6. *PAT14* is not involved in starvation-induced leaf senescence. (A) 3-week-old wild type, *npr1-5*, *NahG*, *pat14-2*, *pat14-2 npr1-5*, and *pat14-2 NahG* plants before 3 days of carbon starvation (D0). (B) 3-week-old wild type, *npr1-5*, *NahG*, *pat14-2*, *pat14-2 npr1-5*, and *pat14-2 NahG* plants after 3 days of carbon starvation (D3 - C).

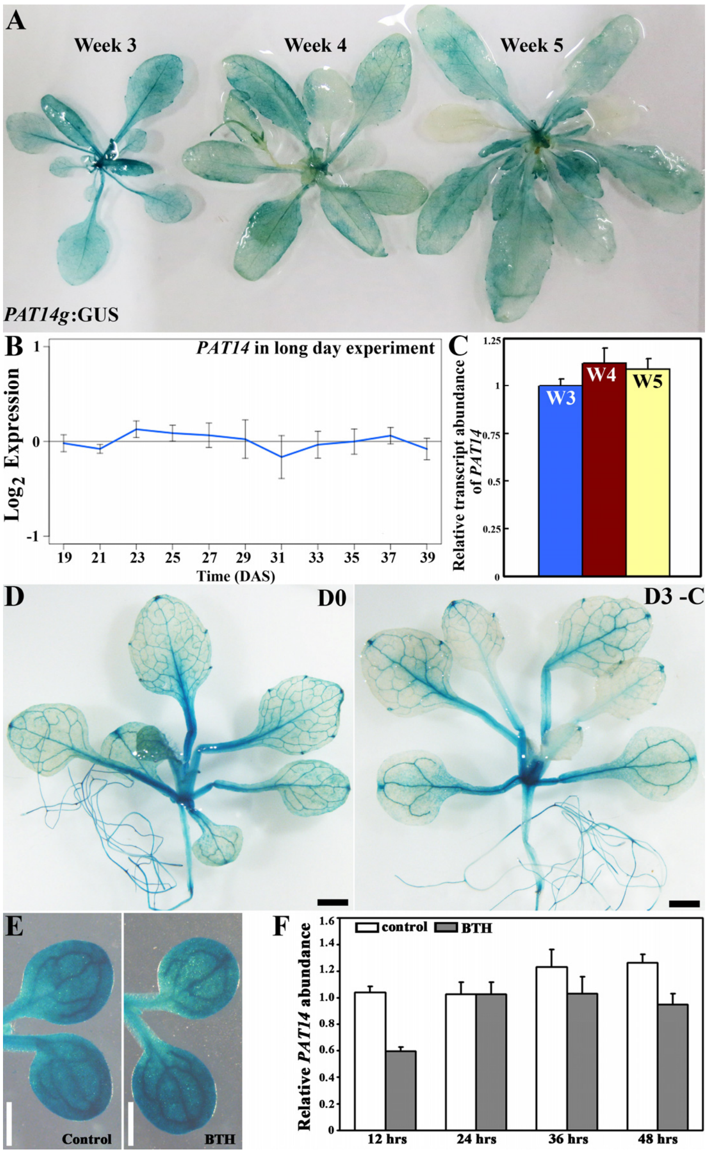

Supplemental Figure 7. Expression of *PAT14* is not responding to age or carbon starvation. (A) Representative histochemical analyses of *PAT14g*-GUS;*pat14-2* transgenic lines at 21 DAG (Week 3), 28 DAG (Week 4), or 35 DAG (Week 5). (B) Expression of *PAT14* in a time-series microarray study of natural senescence (Breeze et al., 2011). (C) *PAT14* expression in leaves at Week 3 (W3), Week 4 (W4), or Week 5 (W5) by quantitative real-time PCR analysis (qPCR). Results are given as means ± standard errors (SEM), N=3. No significant difference was detected (*t*-test, P>0.05). (D) Histochemical analysis of 2-week-old *PAT14g*-GUS;*pat14-2* transgenic seedlings before (D0) and after 3 days in the dark (D3 -C). (E) Histochemical analyses of *PAT14g*-GUS;*pat14-2* transgenic seedling (7 DAG) with mock or BTH treatment for 12 hrs. (F) Expression of *PAT14* in 21 DAG plant leaves upon mock or BTH treatment for 12 to 48 hours by qPCRs. Results are given as means ± SEM, N=3. Bar = 1 mm for (D), 500 μm for (E).

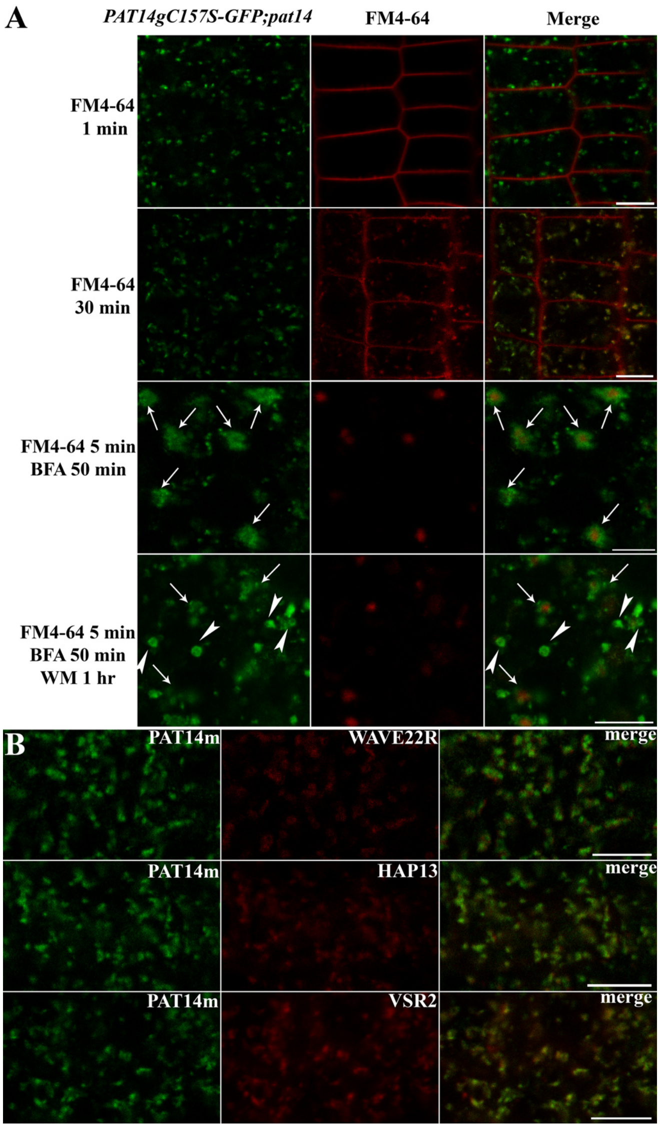

Supplemental Figure 8. Localization of the mutant variant of PAT14, PAT14$_{C157S}$, at the Golgi, the TGN/EE, and PVC/MVB by double-labeling confocal fluorescence microscopy. (A) Confocal fluorescence imaging of *PAT14g*C157S-*GFP* in *pat14-2* (*PAT14g*C157S *-GFP;pat14-2*) stained with FM4-64. Arrows indicate BFA compartments in which both FM4-64 and PAT14C157S-positive TGN/EE accumulates in the center surrounded by PAT14C157S-positive Golgi stacks. Arrowheads indicate ring-shaped PVC/MVB labeled by PAT14C157S upon wortmannin treatment. Bars = 7.5 µm. (B) *PAT14gC157S-GFP* was introduced in transgenic plants expressing RFP-fused markers for Golgi (WAVE22R), TGN/EE (HAP13), and PVC/MVB (VSR2). Partial co-localization was observed for all combinations. Bars = 5 µm.

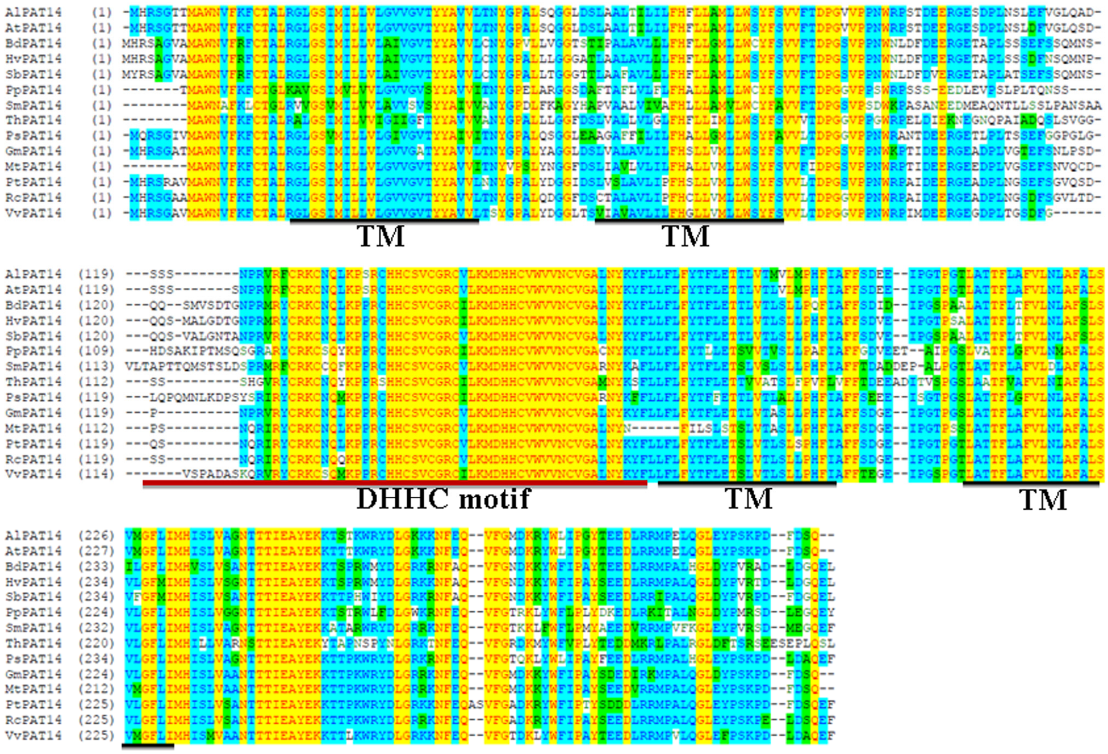

Supplemental Figure 9. Sequence alignment of Arabidopsis PAT14 and its putative homologs from other plant species. Yellow residues are consensus residues while blue or green residues represent residues replaced with other residues of similar characteristics. TMs (for transmembrane domains) and the DHHC motif are underlined. Sequence alignment was performed with Vector NTI (Invitrogen). AtPAT14 homologs can be found at GenBank at the following accession numbers: XP_002878349.1 for AlPAT14, XP_003521795.1 for GmPAT14, XP_002511714.1 for RcPAT14, XP_002320722.1 for PtPAT14, XP_002279896.1 for VvPAT14, XP_003605089.1 for MtPAT14, ABK24332.1 for PsPAT14, XP_002446206.1 for SbPAT14, BAJ93966.1 for HvPAT14, XP_003579517.1 for BdPAT14, XP_002989117.1 for SmPAT14, BAJ34112.1 for ThPAT14, XP_001781101.1 for PpPAT14.

Supplemental Dataset 1. Lists of genes showing at least twofold upregulation (up) or downregulation (down) at transcript abundance in wild type during development (WT5 v.s. WT3) or by *PAT14* loss-of-function (MU3 v.s. WT3).

Supplemental Dataset 2. Enriched GO terms by *PAT14* loss-of-function.

Supplemental Table 1. Primers used in this study.

Usage		No.	5'-3' sequences
PCR	*PAT14*	ZP1147	TCTCATTCCTCAAGCTCCGC
		ZP1771	CGAGAAGGTTTAGTTGATTGCACT
		LB1	ATTTTGCCGATTTCGGAAC
		ZP8	ATATTGACCATCATACTCATTGC
		ZP1203	AAAAACCACTGAGAAATAGCTCCA
RT-PCR	Endogenous	F1	TCTCATTCCTCAAGCTCCGC
	PAT14	R1	TTAGCCTTCCATGGGAAGACATCT
	Exogenous	ZP1599	AGGCACTCTTGCTACTACTTTTCTTG
	PAT14	ZP1600	GCATGGCGGACTTGAAGAAGT
	Zm*PAT14*	ZP11	TGAACTTGTGGCCGTTTACGT
		ZP1506	CACCATGTACAGATCGGCGGGG
	Ta*PAT14*	ZP1628	CTTTAAGAAGGAGCCCTTCACC
		ZP2682	CAACTCTTGCCCGTCCAA
qPCR	*SAG12*	ZP3139	CTATCAAGAATCAAGGCAGCTGCGG
		ZP3140	CGCAGTATCCATTAAACCGCCTTCG
	SAG13	ZP1581	TTGCCCACCCATTGTTAAA
		ZP1582	GATTCATGGCTCCTTTGGTT
	SAG21	ZP1498	GAGATTAGTGGGTAATGATCTG
		ZP1499	AGTTAAGAAGTTCTGAAATGGT
	PAD4	ZP2190	CTTGCCAGTCACCGGAGATGTT
		ZP2191	CGGTTGAATGGCCGGTTATCACC
	SID2	ZP2319	GAGACTTACGAAGGAAGATGATGAG
		ZP2320	TGATCCCGACTGCAAATTCACTCTC
	PDF1.2	ZP2304	TCATGGCTAAGTTTGCTTCC
		ZP2305	ATACACACGATTTAGCACC
	PR1	ZP1583	GTGCCAAAGTGAGGTGTAACAA
		ZP1584	CGTGTGTATGCATGATCACATC
clone	*YUC6*	ZP1399	CACCATGGATTTCTGTTGGAAGAGA
		ZP1400	GATTTTTTTACTTGCTCGTCTT
	PAT14g	ZP1262	CACCCTTCACTAGCTCGGATTTCTCTTTACTCT
		ZP1263	CTGGGAATCAAAGTCGGGTTT
	ZmPAT14	ZP1506	CACCATGTACAGATCGGCGGGG
		ZP1509	AAATCTGGTCTCACTGACAGGATAATC
	TaPAT14	ZP2681	CACCATGCACAGATCGGCGG
		ZP2682	CAACTCTTGCCCGTCCAA
